# Use of digital technology to give and receive feedback in clinical training: a scoping review protocol

**DOI:** 10.1186/s13643-022-02151-8

**Published:** 2022-12-13

**Authors:** Veena S. Singaram, Chauntelle I. Bagwandeen, Reina M. Abraham, Sandika Baboolal, Dumisa N. A. Sofika

**Affiliations:** 1 Clinical and Professional Practice, School of Clinical Medicine, College of Health Sciences, University of KwaZulu-Natal, Durban, South Africa; 2 Discipline of Public Health, School of Nursing and Public Health, College of Health Sciences, University of KwaZulu-Natal, Durban, South Africa; 3 NHS Foundation Trust, Moorfields Eye Hospital, London, UK; 4 Hillingdon Hospitals NHS Foundation Trust, London, UK

**Keywords:** Feedback, Feedback tools, Digital learning, Clinical training

## Abstract

**Background:**

Feedback is vital to improving trainee competencies in medical education. The challenges of the global COVID-19 pandemic related to social distancing to curb the spread of the virus ignited a rapid transition to online medical education. These changes highlight the need for digital feedback tools that enhance the efficacy and efficiency of feedback practices. This protocol is for a scoping review that aims to identify the different digital tools and applications in medical education as reported in the literature, as well as highlight gaps in the current literature and provide suggestions for future technological developments and research.

**Methods and analysis:**

A review of the relevant literature will be guided using the Joanna Briggs Institute methodological framework for scoping studies. Using the search strategy developed by the authors, an electronic search of the following databases will be conducted: PubMed/MEDLINE, EBSCOhost (academic search complete, CINAHL with full text) Scopus, Google Scholar, Union Catalogue of Theses and Dissertations (UCTD) via SABINET Online and World Cat Dissertations and Theses via OCLC. Studies will be identified by searching literature from January 2010 to date of review. Using a validated data extraction form developed for the scoping review, the review team will screen eligible studies and import them onto an electronic library created specifically for this purpose. Data collection for the review will be documented through a PRISMA-P flowchart, and the scoping review will use a basic descriptive content analysis to analyse and categorise the extracted data. All review steps will involve two or more reviewers.

**Dissemination:**

The review will provide a comprehensive list of digital tools and applications used to enhance feedback in clinical training and inform future technological developments. The findings will be disseminated through medical education conferences and publications.

**Supplementary Information:**

The online version contains supplementary material available at 10.1186/s13643-022-02151-8.

## Background

Ensuring competent undergraduate and postgraduate medical students requires a far-ranging multimodal approach to teaching and learning. This includes, but is not limited to, clinical bedside teaching, self-directed learning, didactic input, and feedback about performance. Although the importance of feedback in clinical medical education has been highlighted as far back as the 1970s in the seminal paper by Ende [1], there still needs to be agreement regarding the “ideal model for the delivery of feedback” [2]. A comprehensive definition of feedback, synthesised from the literature, could be described as “a process whereby the desired standard of proficiency in a task has been clearly established. This standard has been communicated to the student. Gaps in performing the task or level of knowledge are identified, based on actual observation of the student, and the student made aware of his or her shortcomings, together with a plan to improve performance” [3, p. 118]. The theory of deliberate practice postulates that a self-reflective feedback loop is critical for the development of expertise instead of just performing a task repetitively until mastered [4].

Feedback from supervisor to novice is provided in multiple forms in different settings, informally at the bedside, the skills lab, in operating theatres, or more formally in scheduled review assessment. However, in keeping with rapid technological development and the ubiquitous availability of “smart” devices, quick feedback using digital tools is increasingly coming to the fore. This development was accelerated by the advent of the COVID-19 pandemic, given the implementation of social distancing practices, which forced a rapid transition to blended teaching and learning, with a greater need for technologically enhanced methodologies. The historical model of teaching in clinical medicine, namely of apprentice observing the master and learning experientially, which to a considerable measure is still prevalent albeit in a more sophisticated form, meant that contact sessions, whether in a lecture hall or in a hospital, had to be mainly abandoned and alternate models quickly developed. Within these simultaneous paradigms, fuelled by this urgent need, it was appropriate to transition from this traditional model which incorporated the practise of “see one, do one, teach one”, to the provision of feedback through more technologically relevant methods. In this study, digital feedback technology refers to devices and electronic formative feedback tools that generate, store, or process feedback data. Ownership of digital devices such as smartphones and tablets is increasing amongst students. Hence, millennials expect to have additional learning opportunities via web‐based and interactive resources [5]. Handheld computing devices have also been used increasingly in clinical settings [6]. Thus, the opportunity presents itself for incorporating such resources into both providing feedback to trainees by supervisors, as well as receiving feedback about such feedback in return — a closing of the feedback loop.

A plethora of feedback tools are described in the literature. While these tools correspond to the wide range of phenomena that the term “feedback” is used to describe in medical education [7], they can differ in the scope of information about clinical performance in specific medical disciplines, as well as their format (whether they are provided in traditional paper-based form or through digital or technological means). For example, in the surgical disciplines, the Ottawa Surgical Competency Operating Room Evaluation (O-SCORE) and System for Improving and Measuring Procedural Learning (SIMPL) have been widely used to guide feedback to residents [8, 9]. In contrast, the Mobile Medical Milestones Application (M3App) has been applied in several family medicine programmes to facilitate giving and receiving feedback [10]. Recently, self-assessment in the form of Entrustable Professional Activities has come to the fore [11]. The advantages and potential benefits of using digital tools have been described from the perspectives of both faculty and students. For example, a study evaluating the M3App in North Carolina, USA, found that medical doctors in postgraduate programmes perceived both the quality and timeliness of feedback to be improved. At the same time, faculty reported increased familiarity with designated milestones [10]. Thus, the use of any particular tool may differ depending on the setting, where the homogeneity of the student population, resource constraints, and availability of relevant supporting infrastructure, amongst other factors, may influence practical applicability. Furthermore, although providing feedback using digital technology can change undesirable habits, the durability of these changes is uncertain as the impact of feedback needs further exploration [12].

This scoping review aims to explore the available evidence about the digital tools used to facilitate feedback practices in clinical training. The study will describe the use of digital technology in giving and receiving feedback in undergraduate and postgraduate medical education across multiple settings and medical disciplines. It will also inform future technological developments and adoptions, current practices, expose existing gaps in knowledge, and justify research to address these gaps.

## Methodology

A scoping review of peer-reviewed and grey literature on digital/electronic feedback tools in medical clinical education will be conducted. The scoping review will be guided by the Joanna Briggs Institute (JBI) framework through its use of the Preferred Reporting Items for Systematic Reviews and Meta-Analyses extension for Scoping Reviews (PRISMA-ScR) as a reporting guide for the review [13].

### Stage 1: Identifying the research question

The main objective of the review is to consider what the available evidence is with regard to the different digital tools and applications being used to enhance the giving and receiving of feedback in undergraduate and postgraduate clinical training. Underpinning this objective are questions about the constraints and facilitators to developing, implementing, and assessing digital feedback provision tools in medical education.

Based on the objectives of this scoping review, we have developed the following research questions.

1. What digital tools and applications are available for giving and receiving feedback in the clinical training environment?
2. What are the main functions or features of the digital tools and applications?
3. How are the digital tools and applications currently being used for feedback in the clinical training environment?
4. What are the barriers and facilitators of using technology to encourage or enhance feedback culture in the clinical environment?

### Stage 2: Identifying relevant studies

Peer-reviewed journals will be reviewed for primary studies with a clear empirical base utilising qualitative, quantitative, and mixed methods addressing the research question. An electronic search of the following databases will be conducted: PubMed/MEDLINE, EBSCOhost (academic search complete, CINAHL with full text) Scopus, Google Scholar, Union Catalogue of Theses and Dissertations (UCTD) via SABINET Online and World Cat Dissertations and Theses via OCLC. Studies will be identified by searching literature from January 2010 to date. A manual search through the main published texts used in medical education teaching and practise will also be conducted. In addition, articles will be searched through the “cited by” search as well as citations included in the reference lists of included articles. The search terms will include e-learning, mobile applications, Google Forms, web-based, telemedicine, smartphones, Twitter, feedback in clinical and medical undergraduate and postgraduate training. Boolean terms (AND) will be used to separate the keywords, and Medical Subject Headings (MESH) terms will also be included during the search. The syntax will be modified where needed. Medical education journals will be searched (i.e. *Academic Medicine*, *Advances in Health Sciences Education*, *BMC Medical Education*, *Journal of Continuing Education in the Health Professions*, *Medical Education*, *Medical Teacher*, and *Teaching and Learning in Medicine*), with the same keywords and date range. Reference lists of selected articles will also be searched for other articles of interest. The services of an experienced subject librarian will be used to ensure that a robust review search strategy is followed. The search strategy will be piloted to check the appropriateness of selected electronic databases and keywords as illustrated in Table 1 (see supplementary material). To compile all relevant evidence sources, identify and remove duplicate records; EndNote X9 reference manager will be used. The review team will search for the evidence sources and import them onto an EndNote library created for this review.

### Stage 3: Study selection

Eligibility criteria will be developed to ensure specific information relating to the research question is included in the studies.

#### Inclusion criteria

For studies to be included, they should meet the following criteria:

- Be available in full text
- Must include medical and/or postgraduate clinical medical education
- Must focus on digital and other forms/modalities/methodologies of electronic feedback
- Since most literature about digital tools for clinical training has been in the recent decade and due to ongoing advances in technology, this review will focus on the latest technologies reported in studies published between January 2010 to date of review.

#### Exclusion criteria

Studies will be excluded should they as follows:

- Not be available in full text
- Focus on feedback in other fields beyond medical education
- Not include detail of digital technologies used
- Only report on the technical specifications of the feedback tool
- Be outside the identified search period
- Not be available in English

#### Eligibility criteria

Eligibility criteria for the scoping review will draw from the JBI mnemonic for the formulation of scoping review questions describing the population, concept, and context (PCC) of the study [14].

##### Population

The scoping review will source all relevant peer-reviewed and grey literature that takes as its objective and the study of development, implementation and assessment of digital feedback provision tools in medical education. The population sample for the review will be undergraduate and postgraduate medical students who participate in the various sourced studies under the review. The rationale for the inclusion of medical students in the study population relates specifically to the importance of their perceptions regarding the use of digital feedback tools in the clinical environment.

##### Context

The context of the study is in the field of clinical practice in medical education and training; however, geographically, the review will source studies and grey literature from around the world in order to develop a most comprehensive appraisal of the development, implementation and assessment of digital feedback tools in the field of clinical practice amongst undergraduates and postgraduate students in medical education and training. By conducting an expansive search, the review can widen its references in terms of meaningfully categorising the nature and typology of digital feedback provision tools in clinical practice in medical education and training.

##### Language

The review will source English language studies only.

##### Date

The date search range for the review will take the period between 2010 and the current review date, which is a period that has seen the most rapid advance in smartphone and digital technologies, with smartphones being described as having been the barometers of change during this period [15]. Not only there has been rapid advancements in the design and use of smartphone digital technologies but also there has also been a rapid increase in terms of accessibility to smartphone devices [16], which because of their sophistication, have been increasingly adopted into clinical practice by healthcare practitioners and medical students in clinical practice [17].

##### Study designs

All study designs will be considered for the review.

### Stage 4: Charting the evidence

An abstract screening tool using Google Forms will be developed and distributed to the review team. Abstract screening, followed by full article screening, will be conducted, including those articles for which an abstract is not available. As illustrated in Table 2 (see supplementary material), a data charting table will be developed and used to extract background information and process the information from each study selected. To ensure that all pertinent information regarding the relevant aspects of the study is collected, the data charting form will first be piloted and then continually updated as required.

### Stage 5: Extracting the evidence

**Fig. 1:**
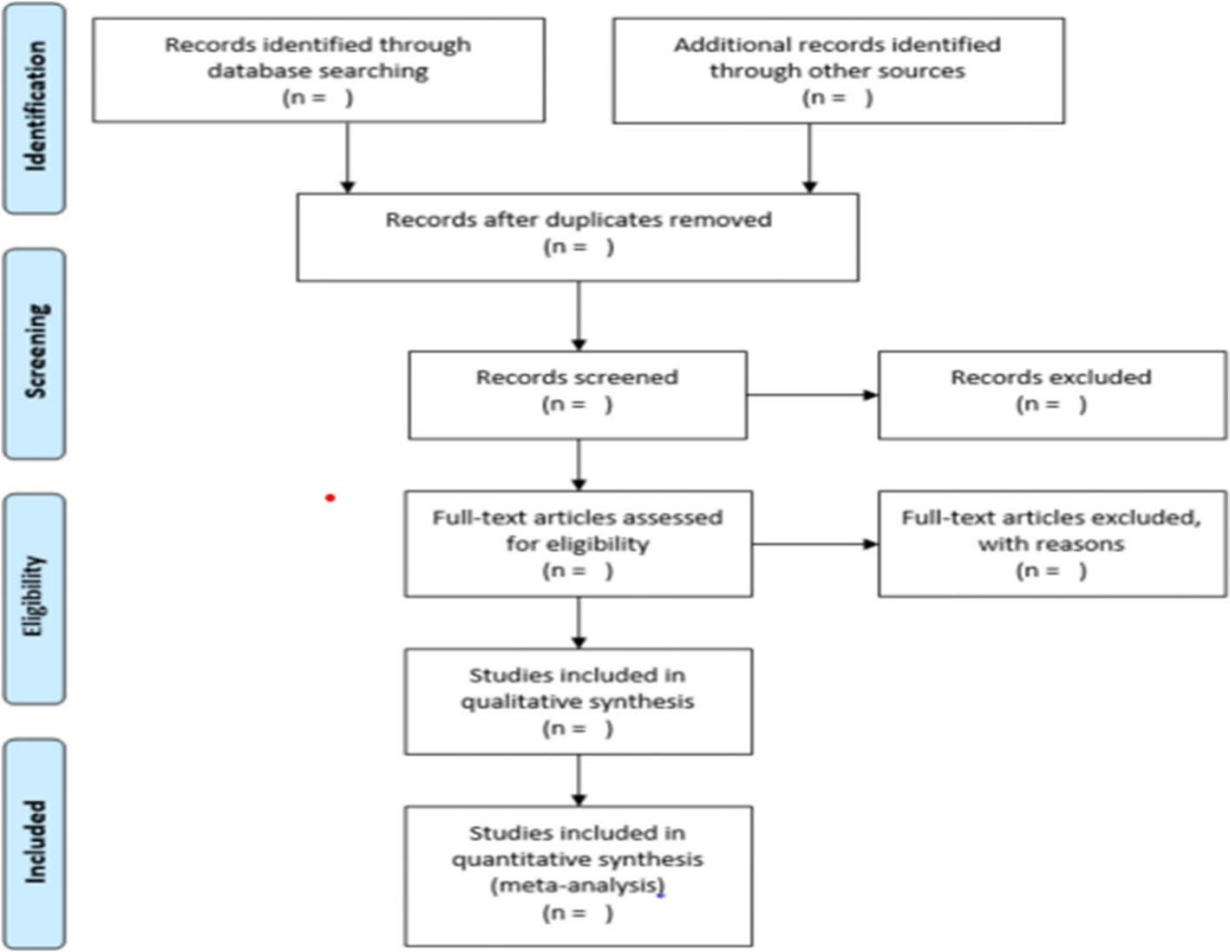
Flow diagram of study selection process based on Preferred Reporting Items for Systematic Reviews and Meta-Analyses guidelines [18]

A data extraction sheet will be constructed via Microsoft Excel. This tool will be designed and piloted by the authors for the use of data extraction as well as data charting by the reviewers. A primary reviewer will use the data extraction tool in consultation with a second reviewer (CB and RA). Information to be extracted from the extraction tool is provided in Table 2 (see supplementary material). A citation manager will be used to create a library for this review. The primary investigator will conduct a search using the key fields in the databases created. Eligible studies will be exported to the citation manager, and duplicates removed before abstracts are screened by two reviewers. Any disagreement will be mediated by a third independent reviewer (VSS). Full article screening guided by the eligibility criteria will then be carried out independently by the review team. Data collection for the review will be documented using a Preferred Reporting Items for Systematic Reviews and Meta-Analyses Protocol (PRISMA-P) flowchart as in Fig. 1.

### Stage 6 Collating, summarising and reporting the results

The summarisation and reporting of the data will use a basic descriptive approach [14] in the form of a content analysis [19]. The basic content analysis will categorise the different digital tools and applications currently being used for feedback in clinical training environment into their uses, into their main features and functions, into their typologies, and into the various constraints and facilitators that characterise their development, implementation and assessment.

## Discussion

In the clinical setting, medical education has moved away from the apprenticeship model developed in the time of Hippocrates. In keeping with the multimodal approach to managing patients, medical students require an acceptable standard of practise that they can strive to achieve, with the necessary interventions implemented timeously by teachers to rectify deficiencies. Feedback that is timeous, comprehensive, directed at the task at hand and feeds forward is vital to improving competencies at both undergraduate and postgraduate medical training programmes. Alternatives to the feedback tools presently employed that can complement and enhance conventional approaches need to be explored in this digital age, where feedback can be made readily and easily available using the device on hand. The global COVID-19 pandemic, which has necessitated social distancing, has resulted in a proliferation of online digital modes of social interaction. There exists an immense application within the medical education platform to explore this digital shift and innovation and capitalising on how it will shape our teaching methods and tools.

This study aims to develop a protocol to review the available literature on the present availability and use of digital feedback tools in the clinical medical education setting. It also has a long-term aim of making implementation recommendations for a comprehensive digital tool to enhance the giving and receiving of feedback in clinical training so as to positively impact on attainment of the desired competencies.

## Supplementary Information

**Additional file 1. **Scoping review response table.

**Additional file 2: Table 2.** Pilot database search results.

## Data Availability

All data generated or analysed during this study will be included in the published systematic scoping review article and will also be available upon request.
