# Supplementary material for: Use of digital technology to give and receive feedback in clinical training: a scoping review protocol
Source: Syst Rev. 2022 Dec 13;11:268. doi: 10.1186/s13643-022-02151-8 (PMC9746573; doi:10.1186/s13643-022-02151-8)
Supplement: Supplementary file 2 — Additional file 2: Table 2. Pilot database search results. [file 13643_2022_2151_MOESM2_ESM.docx]

**Table 2:** Pilot database search results

| **Keyword search** | **Date of search** | **Search engine used** | **Language** | **Number of publications retrieved** |
| --- | --- | --- | --- | --- |
| MeSH terms: undergraduate medical education, clinical training, telemedicine |  | PubMed/MEDLINE | English |  |
| Medical AND students; online AND feedback |  | Ebscohost | English |  |
| Medical student, “online feedback, web-based apps |  | Google Scholar | English |  |
| Telemedicine, smartphones, smart devices, digital technology, undergraduate AND medical students |  | UCTD | English |  |
| Clinical training, medical training, undergraduate AND postgraduate web-based training, feedback tools, digital innovation |  | OCLC | English |  |
