# Supplementary material for: Use of digital technology to give and receive feedback in clinical training: a scoping review protocol
Source: Syst Rev. 2022 Dec 13;11:268. doi: 10.1186/s13643-022-02151-8 (PMC9746573; doi:10.1186/s13643-022-02151-8)
Supplement: Supplementary file 1 — Additional file 1. Scoping review response table. [file 13643_2022_2151_MOESM1_ESM.docx]

Scoping review response table

Thank you for the valued comments. Please see responses in the table below.

| Reviewer #1 | Response |
| --- | --- |
|  |  |
| The background needs to be more succinct. | Background has been changed as suggested. Please see pg. 1-2 |
| Table should be placed at the end of the manuscript as per guidelines of the journal. | Table attached as a supplementary appendix |
| In methodology, it is confusing to have run the search in May 2021 whereas this is a publication of the protocol only | Search dates and results have been removed in keeping with the review only being a protocol |
| I would amend the search period to end in 2022 rather than 2020. In this way you may exclude a significant amount of articles related to virtual learning as a results of the pandemic | Search period amended to end in 2022 (or date of current review) please see pg 9 |
|  |  |
| Reviewer #2 |  |
|  |  |
| Dear authors,  Thank you for your submission. Apologies for the delay, it took some time to secure reviewers for your manuscript. As an Associate Editor, I have acted as the second reviewer. I provide the following comments which I hope you find helpful in revising your paper. My main concern is the need to follow Scoping review methodology more accurately; presently there is a blurring of scoping review and effectiveness review methods which requires attention. | Thank you for these insightful comments.  Scoping review aim, research questions and methodology has been amended as per updated recommended guidelines. |
| The text of the abstract is not in line with the text in the main manuscript. For example, in the abstract you state: ‘This scoping review aims to explore the effectiveness of current digital tools used to give and receive feedback.’ But in the paragraph before the methodology section, you state; ‘This scoping review aims to explore the available evidence about the development, implementation, and evaluation of digital tools used to facilitate feedback practices in clinical training.’ Exploring effectiveness does not lend itself to scoping review methodology, rather a systematic review of quantitative studies (i.e., RCTs) is required. Please revisit and revise the abstract to be in line with the main paper, and with scoping review methodology. | Abstract and main text of the manuscript have been revised. References to effectiveness of digital feedback provision tools amongst undergraduate and postgraduate medical students in the clinical training environment have been removed throughout the manuscript in order to more accurately reflect scoping review methodology |
| Reference 3 in the Background, needs the page number as this is a quote, i.e., [3, p.x] | Page number added, please see pg 3 |
| Can you explain what you mean by 'where speed and accuracy are of the essence' (Line 56) in the context of the sentence, and 'bits and bytes' are increasingly predominating'; the message or your point here is not clear to me. | The context of the sentence has been clarified and the sentence itself has been considerably revised and clarified. |
| Methodology: I think you should refer early on here to the PRISMA ScR (i.e., scoping reviews extension, [https://jbi-global-wiki.refined.site/space/MANUAL/4688844](https://protect-za.mimecast.com/s/PSjkCY6Xq4CojAo0CY2IJC?domain=jbi-global-wiki.refined.site) as a framework for guiding your protocol development | Addressed in methodology |
| I am not clear why you state 'The review will include a quality assessment…' - quality assessment is not listed in one of the steps you outline below this statement. Furthermore, quality assessment is not usually required in a scoping review, as the aim is to scope and describe literature irrespective of quality which is less important in this context. If you wish to add in a quality assessment of included studies, then you need to add this to the steps and provide a rationale. | Acknowledged. A quality assessment will not be forming part of the scoping review. Reference to quality assessment has been removed from the manuscript |
| The reference you use for your scoping review framework is very dated. There are more recent guidance developments that that you should use; specifically, Chapter 11 of the JBI manual [https://jbi-global-wiki.refined.site/space/MANUAL](https://protect-za.mimecast.com/s/OVVkCZ4Xr5CnXQnmh88bNl?domain=jbi-global-wiki.refined.site) | Acknowledged and addressed. These recent guidance developments have been consulted and have been adopted into a new framework that drives the methodology of the review protocol |
| The research sub-question: what is the effectiveness of these tools in improving the frequency and quality of feedback? Is not appropriate to a scoping review. This would require a separate effectiveness review. The scoping review could identify effectiveness studies and thus act as a pre-cursor to an effectiveness review. Please see Chapter 11, section 11.1.1 of the JBI manual for the indications for scoping reviews [https://jbi-global-wiki.refined.site/space/MANUAL/4687794/11.1.1 Why a scoping review?](https://protect-za.mimecast.com/s/PDd2C1j735fwLqwjfE5jfq?domain=jbi-global-wiki.refined.site) Please revise your review questions so they align with scoping review methodology and are not including questions that require other types of reviews | Acknowledged and addressed. The subquestion relating to the effectiveness of digital tools in improving the frequency and quality of feedback have been removed and this is no longer a question that is part of the broader research question since all references to the measuring or evaluation of effectiveness have been removed from the study. |
| The use of PICO is not appropriate for a scoping review; again, this fits an effectiveness review. You need to use PCC (population, concept and context) as recommended for scoping reviews (refer to chapter 11, especially 11.2 which has details about developing a scoping review protocol, and section 11.2.4 which describes inclusion criteria for a scoping review). Another more recent methodological paper that you should look at it is Recommendations for the extraction, analysis, and presentation of results in scoping reviews, JBI Evidence Synthesis, 2022; doi: 10.11124/JBIES-22-00123 | Acknowledged and addressed. Relevant literature consulted and integrated into the methodology of the scoping review in order to more accurately reflect scoping review methodology |
| Please provide a rationale for why you are limiting your search from Jan 2015? Why not search all? | Acknowledged and addressed. A rationale concerning the search date range has been provided in page 9 of the manuscript |
| I would like to see your search strategy more specifically written and aligned with the PCC inclusion criteria. For example, you write Boolean terms (AND, OR) will be used to separate the keywords. You also provide keywords, and state MeSH will also be used, but I can't see this in your searches described in Table 2. That is Table 2 does not align with what you have in your text. Please develop your search strategy to demonstrate exactly how and what will be separated by 'OR' and then 'AND', and in keeping with what you have described (i.e., narrative and Table information should align). | Search strategy revised to be aligned with PCC inclusion criteria. Search terms more accurately demonstrated |
| The phrase All well-known medical education journals…' should be removed (i.e., well known to who?) | Phrase removed |
| Quality appraisal section; as early comment, what is the value of this in the context of your scoping review? Please provide the rationale. This aligns with the comment about revising your scoping review sub-questions to reflect the need for a scoping review compared to another type of review. | Quality appraisal no longer a part of the scoping review protocol |
| For the charting, Collating, summarising and reporting the results sections of your protocol, I refer again to the paper Recommendations for the extraction, analysis, and presentation of results in scoping reviews, JBI Evidence Synthesis, 2022; doi: 10.11124/JBIES-22-00123 which I suggest you should use to rewrite these sections for better clarity | This section has been further clarified based on the recommended guidelines. Please see pg 6-7 and search strategy document attached |
